# Attentional processing of itch

**DOI:** 10.1007/s00426-017-0878-2

**Published:** 2017-06-24

**Authors:** A. I. M. van Laarhoven, S. van Damme, A. P. M. Lavrijsen, D. M. van Ryckeghem, G. Crombez, A. W. M. Evers

**Affiliations:** 1 Unit Health, Medical and Neuropsychology, Faculty of Social and Behavioral Sciences, Institute of Psychology, Leiden University, P.O. Box 9555, 2300 RB Leiden, The Netherlands; 2 Leiden Institute for Brain and Cognition (LIBC), Leiden University, Leiden, The Netherlands; 3 Department of Psychiatry, Leiden University Medical Center, Leiden, The Netherlands; 4 Department of Experimental-Clinical and Health Psychology, Ghent University, Ghent, Belgium; 5 Department of Dermatology, Leiden University Medical Center, Leiden, The Netherlands; 6 Centre for Pain Research, University of Bath, Bath, UK; 7 Institute for Health and Behaviour, INSIDE, University of Luxembourg, Esch-sur-Alzette, Luxembourg

## Abstract

Itch is a prevalent somatosensory symptom that can be highly disabling, because it is likely to draw attention and, as a result, may interfere with the performance of daily activities. Yet, research experimentally investigating attention to itch is lacking. In this study we aimed to investigate attentional processing of itch using multiple behavioral attention tasks. Forty-one healthy participants performed (1) a modified Stroop task with itch-related words, (2) a dot-probe task with itch-related pictures, and (3) a recently developed somatosensory attention task in which the effect of experimentally induced itch on the localization of visual targets was examined. Additionally, a number of self-report questionnaires related to somatosensory attentional processing were administered. Results indicated that participants’ attention was biased toward itch-related words and pictures assessed by means of the dot-probe and modified Stroop task, respectively. For the somatosensory attention task, results showed that itch did not significantly influence the allocation of attention. However, when taking into account the time course of attention during the itch stimulus, data suggested that participants tended to disengage attention away during the itch stimulus. This is the first study that indicates an attentional bias for itch, using methods that have previously been validated for other sensations such as pain. In addition, the newly developed somatosensory attention task may reflect the time course of attention toward a tonic itch stimulus.

## Introduction

Itch is an aversive bodily sensation which is perceived on a regular basis by about 14% of the general population, e.g., it is the primary symptom of diverse chronic skin conditions, such as eczema or psoriasis (Matterne et al., 2011). Itch is associated with an immediate urge to scratch, as a result of which it is highly disruptive and strongly affects patients’ quality of life. Because of its aversive and interruptive characteristics (Ikoma, Steinhoff, Stander, Yosipovitch, & Schmelz, 2006), attention likely plays a role in itch processing (Ikoma et al., 2006; Pfab et al., 2008; Van Laarhoven, Kraaimaat, Wilder-Smith, & Evers, 2010). Attention serves as a gatekeeper, processing and prioritizing signals by their relevance or saliency with the function to detect potential sources of harm for our body (Crombez, Van Damme, & Eccleston, 2005; Legrain et al., 2009). Until now, research on attentional processing of bodily threat has mainly been conducted in the context of pain, revealing that attention is typically biased toward pain and pain-related information (Crombez et al., 2005; Crombez, Eccleston, Van Damme, Vlaeyen, & Karoly, 2012; Crombez, Van Ryckeghem, Eccleston, & Van Damme, 2013; Schoth, Nunes, & Liossi, 2012). Although research investigating attentional processing of itch is largely missing (Van Laarhoven et al., 2010), the relevance of attention in the processing of itch is underscored by several findings, including the overlap with pain, for which attention plays an important role.

Itch is, alike pain, an unpleasant somatosensory sensation, serving as a protector against (potential) harm (Ikoma et al., 2006). Moreover, neurophysiologically, pruriceptive nociceptors that process itch, e.g., mechano-insensitive C-fibers (e.g., responsive to histamine) and polymodal C-fibers (e.g., responsive to cowhage), can also respond to pain stimuli (Andersen, Elberling, & Arendt-Nielsen, 2015; Handwerker, 2014; LaMotte, Dong, & Ringkamp, 2014; Schmelz, 2015). Clinically, itch entails some unique phenomena that highlight its signaling of potential bodily threat requiring attention. For instance, itch can spread to other areas on the body through the phenomenon of “referred itch” (Handwerker, 2014; Ikoma et al., 2006). This also links to its “contagiousness”, entailing that observing others scratching leads to itch and scratching in the observer (Holle, Warne, Seth, Critchley, & Ward, 2012; Ikoma et al., 2006; Schut, Grossman, Gieler, Kupfer, & Yosipovitch, 2015). From an evolutionary perspective, these phenomena probably function to alert us to potential spread of pathogenic agents, such as lice (Schut et al., 2015). Studying the role of attention in itch may unravel attentional processing in relation to well-studied somatosensory sensations, such as pain, and findings may in the long term contribute to the improvement of itch treatment.

To conduct attentional research on itch, appropriate experimental paradigms are required. Although such paradigms, e.g., the modified Stroop and dot-probe paradigms (Crombez et al., 2013; Hu, Fan, & He, 2015; Schoth et al., 2012), have been widely used in pain research, attempts to adapt and apply them to itch are scarce. In the modified Stroop task, participants are requested to read aloud the print colors of displayed words, which are pain related or neutral (Crombez et al., 2013). It is assumed that the saliency of pain-related words interferes with responding, with longer latencies being indicative of more pain-related attention. In the dot-probe task, pain-related stimuli (words or pictures) and neutral stimuli are simultaneously presented at different locations of a display, after which one of the stimuli is replaced by a dot. The reaction time (RT) to respond to the location of the dot is measured. Pain-related attentional bias typically results in longer response latencies for dots contralaterally to the pain stimulus location (incongruent trials) and faster responses for dots ipsilaterally to the pain stimulus location (congruent trials) (Crombez et al., 2013). Studies using these tasks in patients with chronic pain generally indicate that patients have an attentional bias for pain-related information, but findings across studies are mixed, as shown in two recent meta-analyses (Crombez et al., 2013; Schoth et al., 2012). This ambiguity may also be related to the use of symbolic stimuli (i.e., words or pictures) as representation of somatosensory sensations (Crombez et al., 2013). More recently, paradigms using actual somatosensory pain stimulation have been developed, generally indicating enhanced attention for phasic pain stimuli (<1 s) (e.g., Spence, Bentley, Phillips, McGlone, & Jones, 2002; Dowman, 2011; Van Damme, Crombez, Eccleston, & Goubert, 2004b; Van Damme, Crombez, & Lorenz, 2007; Durnez and Van Damme, 2017). Although the allocation of attention may be initially directed toward a pain stimulus, when the stimulus is of longer duration (i.e., tonic) allocation of attention is likely not stable over time, and the degree of attentional bias toward a threatening location (e.g., pain) can vary from moment to moment (e.g., Zvielli, Bernstein, & Koster, 2014). This probably also applies to patients’ clinical symptoms, for which reason tonic induction of pain is more ecologically valid when investigating attention allocation during a stimulus.

In the context of itch, the modified Stroop paradigm has been used to investigate attentional bias. Indirect evidence in patients with chronic skin conditions (psoriasis or burn scars) showed that the patients attended more to disease-related words (e.g., skin, pain, scars, bleed, and also scratch and itch) than healthy controls (Fortune et al., 2003; Willebrand et al., 2002). Recently, we developed a modified Stroop task that specifically used itch-related words. An exploratory study in a small group of patients with chronic itch due to burn injury and healthy controls showed that both groups had an attentional bias for the itch-related words (Van Laarhoven et al., 2016). Considering the contagiousness of itch, attention tasks using pictorial stimuli (e.g., dot-probe task) might be very well suitable to measure attentional itch processing. Moreover, given that itch, and particularly clinical itch, often lasts longer than a second, the use of tonic somatosensory itch stimuli seems most representable. However, to the best of our knowledge, no such tasks are available for itch.

The present study investigated attentional processing of itch and itch-related information in healthy volunteers using both traditional attention tasks applied to itch (i.e., the modified Stroop task and the dot-probe task) as well as a newly developed task that makes use of somatosensory itch stimuli (i.e., the somatosensory attention task; SAT). It was hypothesized that participants would display more attention toward itch stimuli (either symbolic representations or somatosensory stimuli) than toward neutral stimuli. Furthermore, we explored the time course of attention allocation during the first and second half of the itch stimuli within the SAT. Finally, we explored the relationship between attentional processing of itch and self-reported attention for bodily sensations, neuroticism, and catastrophizing.

## Methods

### Participants

Forty-one healthy volunteers (32 female/9 male) aged between 18 and 30 years were included in the study (mean age = 21.5, SD = 2.0; range 18.0–28.3 years). Participants were recruited through advertisements at Leiden University and the Leiden University Research Participation system (SONA systems Ltd, Tallinn, Estonia). Inclusion criteria for participation were being aged between 18 and 30 years (to include a homogenous group considering that reaction times increase with age; Woods, Wyma, Yund, Herron, & Reed, 2015) and fluent in Dutch language. Exclusion criteria for participation were severe morbidity (e.g., multiple sclerosis, diabetes mellitus, heart or lung disease, rheumatoid arthritis, vasculitis), psychiatric disorders (e.g., depression), use of pacemaker, chronic itch or pain complaints, current use of medication, color blindness, and pregnancy. All participants were students or had just finished tertiary education. The protocol was approved by the local medical review ethics committee and all participants provided written informed consent.

### Attention tasks

A modified Stroop task, a dot-probe task, and a somatosensory attention task (SAT) for itch were used to measure attentional processing of itch. All tasks were presented using E-prime software (version 2.0, Psychology Software Tools Inc., Sharpsburg, PA, USA) using a Dell optiplex 3010 computer with Philips Brilliance 225 TFT screen (Resolution 1280 × 1024 at 60 Hz). Finger response buttons, attached to the table at a fixed position, were attached to a serial response box (Psychology Software Tools Inc. Sharpsburg, PA, USA).

#### Stroop task modified for itch

A previously developed modified version of the Stroop task was used to measure attentional processing of itch-related and other emotional words (van Beugen et al., 2016; Van Laarhoven et al., 2016). The task included eight words related to itch (itchy, mosquito bite, fleabite, nettle, head lice, itch, louse, scratching), eight neutral words (drinking mug, kettle, nutcracker, refrigerator, kitchen, tablecloth, light bulb, doorknob) as well as eight negative words, eight positive words, and eight words related to stigmatization (van Beugen et al., 2016). Only the itch and neutral category were relevant for the present research design and therefore reported here. The words related to itch had been validated, along with other word categories, in a pilot study by a group of 43 dermatology patients, healthy participants, and health professionals (see van Beugen et al., 2016). In this pilot study, the itch words were selected based on high applicability to itch and a slightly negative valence. The words in the other categories have also been used in our previous study (van Beugen et al., 2016) and were taken from the Dutch Emotional Word list (Arnold et al., 2011). All words were single words in Dutch and matched in length between categories. Each word category consisted of 40 words (8 words repeated 5 times) in different colors (i.e., white, green, blue, yellow, red) that had been randomized in advance (for each card the same order of colors); the background was black. The words of one category were displayed at once in random order (randomized by E-prime) on the computer screen (block-design). Participants were instructed to name aloud the print color of the words displayed, as quickly and accurately as possible. The card was displayed up until the participant finished naming aloud the colors of the displayed words and time was measured. No maximum time limit was determined in advance. However, participants’ performance was monitored by the test leader. The performance level of all participants was considered satisfactory.

#### Dot-probe task for itch

A dot-probe paradigm (e.g., Crombez et al., 2013) modified for itch was used to measure attention bias for itch-related pictures. For this task, ten itch pictures and ten neutral pictures were used. Itch pictures had been validated in the same pilot study as the itch words, with respect to the applicability on itch and a slightly negative valence (van Beugen et al., 2016). The neutral pictures were selected from the International Affective Picture System (IAPS) database, where they had been validated as neutral (numbers 7004, 7006, 7010, 7025, 7035, 7053, 7080, 7150, 7175, 7705) (Lang, Bradley, & Cuthbert, 2008) and matched as much as possible with the itch pictures with regard to complexity and color. Additionally, four pairs of neutral pictures (IAPS numbers 7000, 7002, 7009, 7090) were used for practicing purposes. For every trial, first a fixation cross was displayed for 500 ms in the middle of the computer screen. Thereafter, a pair of pictures (in randomized order) was displayed side by side on the screen for 500 ms, which display time has most commonly been used (Crombez et al., 2013; Schoth et al., 2012), followed by the presentation of a dot (probe), replacing one of the two pictures, for at maximum 2000 ms (i.e., response window). The width of all pictures was 11.5 cm on the screen, with most picture pairs presented in landscape format (height varying between 7.6 and 9.0 cm) and two pairs in portrait format (height 13.2 and 13.8 cm). The shape of both pictures within one pair always matched. The pictures were placed in the middle of the screen height and on 25 and 75% of the screen width leaving 6.8 cm between the two pictures. The size of the dot was 0.5 cm. Localization of the dots required attentional orienting. Participants were required to respond as quickly as possible to the location of the dot (left/right), by pressing (with the index finger) the corresponding response button. Upon responding, the dot disappeared. The interval in-between trials was 100 ms. Test trials consisted of sequentially displaying 40 pairs of an itch-related and a neutral picture. The target pictures as well as the probe were presented equally often at the left or right position of the screen and the dot probe was equally likely to replace either an itch picture or neutral picture. Replacement of the itch picture by the dot is referred to as “congruent trials”, whereas replacement of the neutral picture is referred to as “incongruent trials”.

#### Somatosensory attention task

**Fig. 1:**
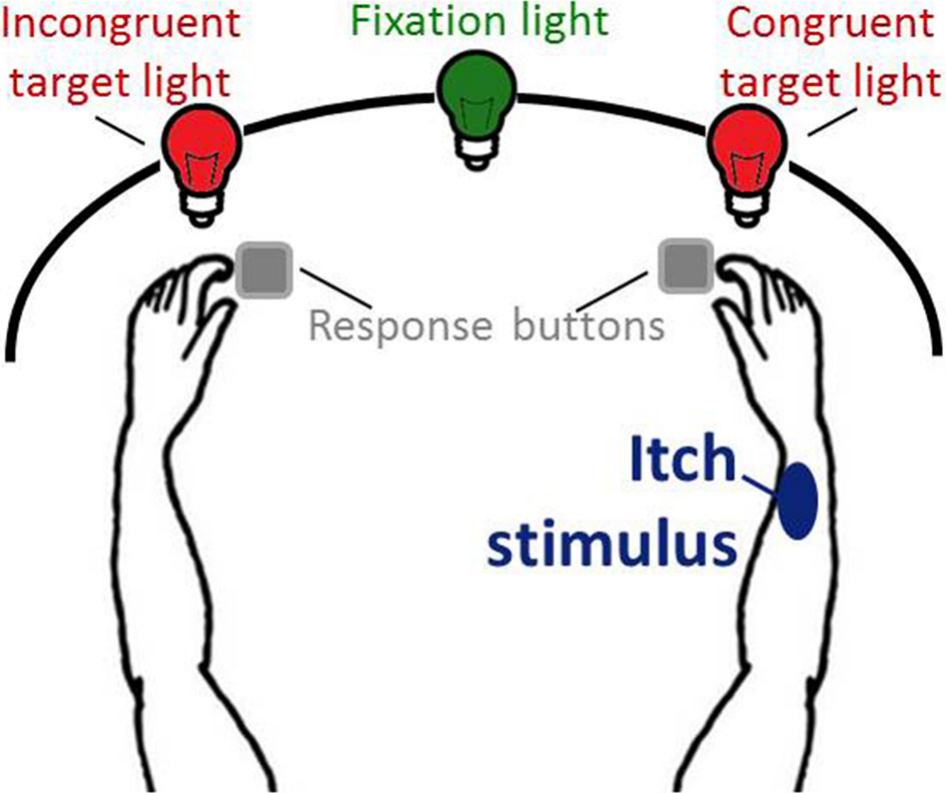
Schematic representation of the setup of the somatosensory attention task. The side of itch stimulation was randomized across participants, and in this example the itch stimulus is given on the right arm. During a trial, first the fixation light is turned on for 1000 ms, whereafter one of the target lights is turned on for 200 ms. Therefore, at any time, either no or a single light is turned on. Participants’ response buttons to respond to the target lights are located on a platform right below both target lights

The somatosensory attention task (SAT) was used to measure attentional processing of somatosensory itch stimuli (see Fig. 1 for a schematic representation of the setup). This task was based on the cross-modal attention task for pain-related attention developed by Van Damme et al. (2007). However, tonic itch stimuli were used in the SAT as the on- and offset of the itch sensation after phasic stimuli cannot be reliably predicted, as itch is often delayed. More importantly, tonic itch stimuli better represent clinical itch. A black curved screen of ca. 50 cm height was placed in front of the participant. There were three LED lights in the screen at circa 10 cm height. The middle, green LED was the fixation light, while two red LEDs, attached approximately 25° to the left and right from the middle LED, functioned as target lights. Right below the left and right LED, there was a platform on which a left and right response button, respectively, was attached.

Itch was induced by electrical stimulation, delivered by a constant current stimulator (Isolated Bipolar Constant Current Stimulator DS5, Digitimer, United Kingdom) (see also Bartels et al., 2014). According to a standardized protocol, which was previously developed with the aim of inducing substantial levels of pure itch in a large proportion of people (e.g., Bartels et al., 2014; Van Laarhoven et al., 2016), electrodes were attached to the inner side of the wrist through two surface electrodes (a disk electrode of ø 1 cm and a reference electrode of ø 2 cm, VCM Medical, Leusden, the Netherlands). Electrodes were attached unilaterally (randomized based on hand dominancy) to minimize crossover effects between itch blocks and control blocks, as itch takes considerable time to disappear after stimulus termination (e.g., Papoiu, Tey, Coghill, Wang, & Yosipovitch, 2011). According to our standard protocol (Bartels et al., 2014; Van Laarhoven et al., 2016), the itch stimuli were delivered at 50 Hz frequency, 0.1 ms pulse duration, 0.05 mA/s ramping, and at a maximum current intensity of 5.00 mA. Practice trials for familiarization with the electrocutaneous stimuli consisted of two measurements from 0.01 mA up to the intensity at which the participants indicated the moment that they experienced a sensation for the first time and the first moment they felt some itch. The intensity of the itch stimuli for the SAT was individually tailored on the basis of two measurements of the itch threshold “the first moment you feel the urge to scratch”, previously shown to induce itch levels of ≥2 on a scale from 0 (no itch) to 10 (worst itch ever experienced) in over 90% of participants (Bartels et al., 2014). To ascertain that the participants felt itch during the attention task, the average current intensity of the itch thresholds was the start intensity of the 35-s itch stimuli used before (i.e., baseline stimuli) and during the SAT. Given the continuously ramping of 0.05 mA/s, the end current intensity was the start intensity plus 1.75 mA. In the case the electrical current would exceed 5.00 mA, the stimulation started at 3.25 mA and ended at 5.00 mA. In this study, the mean start intensity was 2.11 mA (SD = 0.83). The level of experienced itch during the SAT was scored on a numerical rating scale (NRS) ranging from 0 (no itch) to 10 (worst itch ever experienced).

The SAT consisted of eight blocks of 35 s each, of which four blocks had itch stimuli (itch blocks) and four blocks were without itch stimuli (control blocks). Within each 35-s block, there were ten trials in which first the fixation light (green LED light) was turned on for 1000 ms, extinguished, and then either the left or right target (red LED light) was turned on for 200 ms. The response window for pressing a button was 1500 ms (based on Van Damme et al., 2007). Over all blocks, half of the visual targets were presented at the body side where the electrodes were attached (“congruent trials”) and half of the visual targets were presented at the opposite side (“incongruent trials”). Within a single block, the ten targets were given in random order (congruent or incongruent), either in 5/5, 4/6 or 6/4 ratio to maximally avoid the predictability of the target location. Also, the time interval after a target before the onset of the next fixation light was random and varied between 1000 and 2500 ms. E-prime randomized the order of the eight blocks per participant without restrictions (resulting in 3 participants having all itch blocks at the end and 3 participants having all itch blocks in the beginning). Participants were not aware of the number or distribution of itch and control blocks. The standard interval between blocks was 1 min, which was extended by 1 min up to a maximum of 5 min interval in the case the NRS itch exceeded 1.0 [mean interval duration = 1.4 (SD = 0.92) and 1.1 (SD = 0.40) min, during itch and control blocks, respectively].

### Self-report questionnaires

Participants completed a battery of validated self-report questionnaires. All were administered using the online system Qualtrics (Provo, Utah, USA).

The presence of physical symptoms during the past 2 weeks was assessed by the two visual analog scales (VAS) ranging from 0 (no itch/pain) to 10 (worst itch/pain experienced) for itch and pain from the Impact of Chronic Skin Disease on Daily Life (ISDL) health status inventory (Evers et al., 2008).

Psychological distress was measured via the Hospital Anxiety and Depression Scale (HADS) (Zigmond and Snaith 1983). The Cronbach alpha was 0.63 for the subscale depression and 0.71 for the subscale anxiety.

Neuroticism was measured with the Eysenck Personality Questionnaire revised short scale (EPQ-RSS) (Sanderman et al., 1995), from which the total score of the neuroticism subscale (Cronbach alpha 0.78) was calculated.

Attentional focus on bodily sensations was measured using the Body Vigilance Scale (BVS) (Schmidt, Lerew, & Trakowski, 1997), the Body Sensations Questionnaire (BSQ)-frequency version (De Ruiter, Garssen, Rijken, & Kraaimaat, 1989; Arrindell, 1993) similarly to our previous study (Van Laarhoven et al., 2010). Additionally, due to the lack of questionnaires focusing on itch-related attention, we adjusted the Pain Vigilance and Awareness Questionnaire (Roelofs, Peters, McCracken, & Vlaeyen, 2003) for use in itch by substituting the word “pain” by “physical sensations” for all concerned items (PVAQ-A). The Cronbach alpha of the BVS and BSQ in the present study was 0.59 and 0.81, respectively. For the PVAQ-A, adjusted to physical sensations, Cronbach alpha was 0.87.

Catastrophizing about physical sensations was measured using the Pain Catastrophizing Scale (Sullivan, Bishop, & Pivik, 1995), adjusted for physical sensations by substituting the word “pain” for “physical sensations” for all concerned items. The Cronbach alpha for the adjusted pain catastrophizing scale (PCS-A) in the present study was 0.77.

### Procedure

Potential participants were informed about the study by written information and asked to fill in online screening questionnaires (demographics, presence of physical symptoms, EPQ-RSS, and HADS). In the case of uncertainties about eligibility based on the screening questionnaires, inclusion and exclusion criteria were additionally checked by a telephone call. Eligible participants made an appointment for participation. Participants were instructed not to take medication 12 h prior to testing and refrain from intake of alcohol and drugs 24 h before attending the experiment. Upon arrival at the test facility, participants were informed about the procedure and they were told that they were free to terminate the experiment at any time. Then all participants signed the informed consent. Participants rated their current levels of itch and pain on an NRS ranging from 0 (no itch/pain) to 10 (worst itch/pain ever experienced) and filled out the remaining questionnaires assessing individual characteristics related to attentional processing of physical sensations (PCS-A, BVS, BSQ, PVAQ-A).

To prepare for the electrical stimuli during the SAT (see also Attention tasks—Somatosensory attention task), participants held their wrist to be stimulated (randomized either dominant or non-dominant) for 3 min in a warm water bath of ca. 32 °C (Bartels et al., 2014). After attaching the electrodes to the wrist, the participants were familiarized with the practice measurements. Subsequently, the itch threshold was determined twice. Then, the baseline itch stimulus was applied for 35 s, after which the SAT began. Participants’ wrists rested on the platform of the SAT with the index fingers of both hands positioned on the left and right response button, respectively. Participants were instructed to respond as quickly as possible to the location of a lightened target LED, by pressing the button congruently to the side of the target. Before each block, participants were informed whether they would receive an itch stimulus (i.e., in an itch block) or not (i.e., in a control block). At the start of each block, the experimenter counted down from 3 to 0, to indicate the start of a block. Directly following each block, participants were asked to report the levels of itch experienced during the block on an NRS ranging from 0 (no itch) to 10 (worst itch ever experienced). After each block, there was a 1-min interval, after which the NRS was asked again. Based on this score, the interval was either extended or not and the next block began. After eight blocks of the SAT, the electrodes were removed. The modified Stroop task and the dot-probe task for itch followed, of which the order was randomized across participants. For the modified Stroop task, participants were instructed to name aloud the print color of the words displayed, as quickly and accurately as possible. The experimenter, blinded to the word category that was displayed, remained in the room and pressed a button after the participant finished naming the colors of all words of one card (to standardize the measurement of finishing a card; Van Beugen et al., 2016), which was recorded by E-prime, and registered the number of mistakes per card (Van Laarhoven et al., 2016). For the dot-probe task, the participants were first instructed how to perform the task in four practice trials with a pair of neutral pictures on the screen after which a dot appeared to which participants responded. When the task was clear to the participant, he/she was left alone in the laboratory to conduct the 40 trials with pairs of itch-related and neutral pictures. After performing all tasks, participants were asked about their expectations and experience of the tests, were given a short debriefing, and received a monetary reimbursement.

### Statistical analyses

First, data of the dot-probe task, modified Stroop task, and SAT were extracted from E-prime. For the dot-probe task, RT was excluded when <150 or >1500 ms (0.2% of the RT) and when responses were incorrect (3.7% of the RT) (based on Van Damme et al., 2007). Also for the SAT, trials with RT < 150 ms (0.2% of the RT) and RT for incorrect responses (0.04% of the RT) were excluded. As the response window for the SAT was already 1500 ms, there was no additional cutoff for maximum RT. The SAT data of 34 participants were used since SAT data turned out to be unavailable or insufficient (i.e., ≤70% of adequate SAT data) for 7 participants as a result of technical problems, e.g., a broken electrode (*n* = 1), malfunctioning electrical stimulator and therefore lack of time (*n* = 2), malfunctioning response button (*n* = 1), cables of SAT response buttons inadequately attached to the serial response box (*n* = 2), or network error (*n* = 1). The SAT data were preprocessed using Matlab and Statistics Toolbox Release 2012b (The MathWorks, Inc., Natick, MA, USA) by calculating the mean RT per trial type (congruent and incongruent trials during both itch and control blocks) for each participant as well as the mean RT when blocks were split into two segments of 17.5 s. For each trial type within both segments, the Cronbach’s alpha was calculated.

Statistical analyses were conducted using SPSS 23.0 software (IBM SPSS Statistics for Windows, Armonk, NY, USA). Accuracy (i.e., number of mistakes made) was checked for each task, enabling removing participants making an excessive number of mistakes (i.e., >30%). No subjects had to be removed based on this criterion. Variables were checked for normal distribution and log-transformed whenever needed. Transformation was successful for the majority of the variables except for the RT of the Stroop card for itch, due to an outlier (>3 SD from the mean) and the attentional bias indices for the dot-probe task and the modified Stroop task.

For the dot-probe task, a 2 × 2 repeated measures analysis of variance (RM-ANOVA) was carried out with the presentation side of the dot (left/right) and the position of the itch picture (left/right) as within-subject variables (log-transformed), thereby taking into account potential associative mapping of emotional valence in physical space (Casasanto, 2009). For the modified Stroop task, RT values for the itch and neutral word category (within-subjects factor) were compared in an RM-ANOVA. These analyses were also performed without any outlier. For the SAT, as manipulation check an RM-ANOVA was applied comparing the NRS itch scores in the itch blocks with the control blocks. To test whether RT for congruent and incongruent trials during the itch and control blocks significantly differed, a 2 × 2 RM-ANOVA was carried out with congruency (congruent/incongruent; as opposed to the side of the attached electrodes) and block type (itch/control blocks) as within-subject factors. The main effects of congruency and block type were calculated, as well as the congruency × block type interaction. In addition, to explore the course of attention over time, a 2 × 2 × 2 RM-ANOVA was then conducted, with log-transformed variables, using three factors, i.e., congruency (congruent/incongruent), block type (itch/control blocks) and time (first half/second half of the blocks). The main effects of time and the congruency × block type × time interaction were calculated.

For all the RM-ANOVAs conducted (within-subjects design), a generalized eta squared was calculated (Lakens, 2013). Post hoc analyses were carried out by performing the main analyses for the three behavioral attention tasks while including participant’s sex (centered) as covariate. In additional post hoc analyses, the side of itch stimulation during the SAT (centered) was included as covariate in the main analyses for the SAT and the dot-probe task (performed after the SAT). The split-half reliability of the dot-probe task and SAT was investigated by calculating the Spearman–Brown coefficient, for each trial type separately.

Finally, attentional bias (AB) indices were calculated for the three tasks. For the modified Stroop task, the RT for the neutral words was subtracted from the mean RT for the itch words. For the dot-probe task, the mean RT of the congruent trials was subtracted from the mean RT of the incongruent trials while taking into account the display side of the itch picture (((RT_Incongruentleft_ − RT_congruentleft_) + (RT_Incongruentright_ − RT_congruentright_))/2) (Schoth et al., 2012). For the SAT, the mean RT of the incongruent trials was subtracted from the mean RT of the congruent trials during the itch blocks (RT_incongruentitchblock_ − RT_congruentitchblock_). A positive AB index for all these tasks indicated that attention was biased toward itch. Subsequently, correlation coefficients were calculated between total scores of the self-report questionnaires measuring neuroticism (EPQ-RSS), self-reported attention (BVS, BSQ-f, PVAQ-A), and catastrophizing (PCS-A) and the AB indices of the SAT (Pearson correlation coefficients) and the dot-probe task and modified Stroop task (Spearman correlation coefficients).

## Results

The total scores of self-report questionnaires of the 41 participants are shown in Table 1.

**Table 1:** Total scores of self-report questionnaires (*n* = 41)

	Mean (standard deviation)	Range
Levels of itch at baseline	0.5 (1.0)	0.0–3.5
Levels of pain at baseline	0.0 (0.1)	0.0–0.5
Affect		
Anxiety (HADS-anxiety)	2.4 (0.3)	1.0–2.9
Depression (HADS-depression)	2.6 (0.3)	1.7–3.0
Personality characteristics		
Neuroticism (EPQ-RSS)	3.0 (2.6)	0–10
Attention to bodily sensations		
BVS	11.4 (4.9)	1.8–20.2
BSQ	2.1 (0.5)	1.1–3.1
PVAQ-A	25.5 (10.6)	3–51
Catastrophizing		
PCS-A	9.4 (5.1)	0–23

*HADS* Hospital Anxiety and Depression Scale (theoretical range 0–21 per subscale), *EPQ-RSS* Eysenck Personality Questionnaire revised short scale (theoretical range 0–12 neuroticism subscale), *BVS* Body Vigilance Scale (theoretical range 0–40), *BSQ* Body Sensations Questionnaire (theoretical range 1–5), *PVAQ-A* Pain Vigilance and Awareness Scale, adjusted for physical sensations (theoretical range 0–80), *PCS-A* Pain Catastrophizing Scale, adjusted for physical sensations (theoretical range 0–52)

### Modified Stroop task for itch

With regard to the accuracy during the itch and neutral words of the modified Stroop task, participants made on average 1.0 (SD = 1.1) and 0.7 (SD = 0.9) mistakes, respectively (theoretical maximum 40 per category). Participants needed on average 26.7 (SD = 5.7) and 25.0 (SD = 4.7) s to read aloud the colors of the itch and neutral words, respectively. For the itch words, RT was significantly longer than for the neutral words [*F*(1, 40) = 20.98, *p* < 0.001; $\eta_{\text{G}}^{2}$ = 0.029]. Comparable results were found when removing the one outlier in RT on the itch words.

### Dot-probe task for itch

With regard to the accuracy during the dot-probe task, participants made on average 1.5 (SD = 1.5) mistakes, ranging from 0 to 5 during the complete task (theoretical maximum 40). The mean RTs for the trials per display side of the computer screen are given in Table 2. The 2 × 2 RM-ANOVA indicated a significant interaction effect for the itch picture position (left/right) × dot position (left/right) [*F*(1, 40) = 8.25, *p* = 0.006; $\eta_{\text{G}}^{2}$ = 0.01], with longer RT for incongruent trials (e.g., itch picture right, dot left) than for congruent trials (e.g., itch picture left, dot left). There were no significant main effects of either itch picture position [*F*(1, 40) = 0.02, *p* = 0.90; $\eta_{\text{G}}^{2}$ = 0.0002] or dot position [*F*(1, 40) = 1.68, *p* = 0.20; $\eta_{\text{G}}^{2}$ = 0.005].

**Table 2:** Mean reaction times (in ms) with standard deviation (SD) for the trials of the dot-probe task for itch (*n* = 41) per display side on the computer screen

	Itch picture position, left	Itch picture position, right
	Mean (SD)	Mean (SD)
Dot position, left	307.4 (46.4)	314.9 (36.0)
Dot position, right	322.3 (48.4)	312.7 (47.8)

The split-half reliability analyses showed Spearman–Brown coefficients of 0.82 when both itch picture and dot were shown on the left, 0.67 when the itch picture was shown on the left and the dot on the right, 0.80 when the itch picture and dot were shown on the right, and 0.46 when the itch picture was shown on the right and the dot on the left.

### Somatosensory attention task

During the baseline itch stimulus before the SAT, given at the same intensity of the SAT itch stimuli, participants scored on average NRS itch of 5.0 (SD = 2.4). The manipulation check showed that during the SAT, participants scored higher levels of itch during the itch blocks (*M* = 3.6; SD = 2.2) than during the control blocks (*M* = 0.3; SD = 0.3), which was significant in the RM-ANOVA [F(1, 33) = 77.54, *p* < 0.001; $\eta_{\text{G}}^{2}$ = 0.70]. With regard to the accuracy of responding during the SAT, participants made on average 0.4 (SD = 0.7) mistakes, ranging from 0 to 2 mistakes during the complete task (theoretical maximum 80).

The mean RTs during itch and control blocks for the congruent and incongruent trials are displayed in Table 3. RM-ANOVA comparing the RT for congruent with incongruent trials (factor 1: congruency) during the itch and control blocks (factor 2: block type) did not show a significant main effect of congruency [*F*(1, 33) = 1.10, *p* = 0.30; $\eta_{\text{G}}^{2}$ = 0.003] or block type [*F*(1, 33) = 1.86, *p* = 0.18; $\eta_{\text{G}}^{2}$ = 0.005]. There was also no significant interaction effect of congruency × block type [*F*(1, 33) = 0.97, *p* = 0.33; $\eta_{\text{G}}^{2}$ = 0.001).

**Table 3:** Mean reaction times (in ms) with standard deviation (SD) for the congruent and incongruent trials of the somatosensory attention task (SAT) during itch blocks (itch stimulus) and during control blocks (no itch stimulus) *n* = 34

	Congruent trials^a^	Incongruent trials^a^
	Mean (SD)	Mean (SD)
Itch blocks	454.7 (53.1)	445.6 (52.9)
Control blocks	443.7 (50.4)	442.3 (53.8)

^a^For congruent trials, target lights during the SAT were given at the side where the itch electrodes were attached, while for incongruent trials, the target lights were given contralaterally to the location of the itch electrodes. During itch blocks, itch stimuli were applied, while during control blocks, no somatosensory stimulation was applied

The split-half reliability analyses for the SAT showed Spearman–Brown coefficients of 0.84 and 0.82 for the congruent and incongruent trials during itch blocks, respectively, and 0.90 and 0.86 for trials congruent and incongruent to the electrode location during control blocks, respectively.

**Fig. 2:**
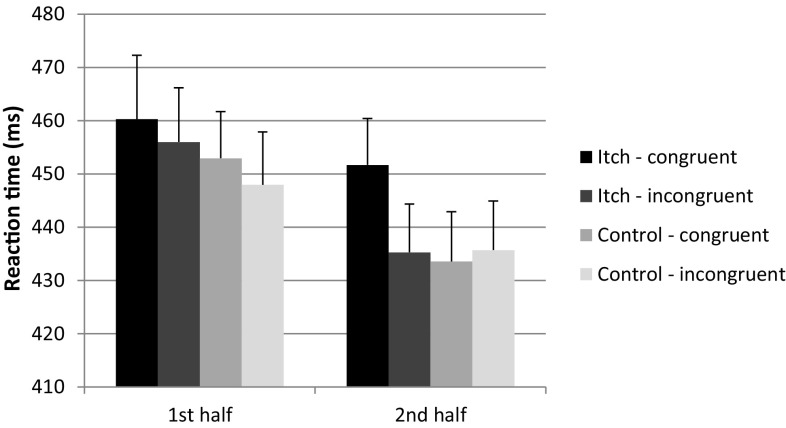
Reaction times (RT) for the different categories of the somatosensory attention task (SAT), i.e., itch or control blocks with congruent and incongruent trials during the first and second half of the 35-s blocks are displayed (*n* = 34). Error bars represent standard errors of the mean (SEM). Post hoc RM-ANOVAs showed a significant interaction effect for congruency × block type (itch versus control) (*p* < 0.05) in the second half of the blocks, which was not the case during the first half of the blocks (*p* = 0.80)

Exploration of the time course of attention is visualized in Fig. 2 by displaying average RT per category during the first and second half of the SAT blocks (*n* = 34). The RM-ANOVA showed a significant main effect of time [*F*(1, 33) = 17.65, *p* < 0.001; $\eta_{\text{G}}^{2}$ = 0.02], indicating that RT decreased over time. There was a non-significant trend for the congruency × block type × time interaction effect [*F*(1, 33) = 3.30, *p* = 0.078; $\eta_{\text{G}}^{2}$ = 0.003]. Post hoc RM-ANOVAs showed that in the second half of the blocks, there was a significant interaction effect for congruency × block type [*F*(1, 33) = 4.34, *p* = 0.045; $\eta_{\text{G}}^{2}$ = 0.02], with profile plots showing that RT was longer for the congruent trials during itch blocks than RT in the other categories, suggesting that during the second half of the blocks less attention was directed to the itch stimulation. This was not the case in the first block, where no significant interaction effect was found for congruency × block type [*F*(1, 33) = 0.063, *p* = 0.803; $\eta_{\text{G}}^{2}$ = 0.0003]. The Cronbach alphas per time segment indicated adequate internal consistency over the four blocks of each trial type as the Cronbach alphas varied between 0.69 and 0.83 for segment 1 and between 0.69 and 0.84 for segment 2.

### Analyses controlled for potential confounders

When controlling for sex in the main analyses, similar results were obtained for the modified Stroop task (significant main effect for word category, *p* < 0.001, with all contrasts displaying significant differences between the itch category and the other categories, all *p* < 0.05), the dot-probe task (significant itch picture position × dot position effect, *p* < 0.01), and the SAT (main effect of block type, *p* = 0.19, main effect of congruency, *p* = 0.31, block type × congruency effect p = 0.34).

When controlling for the side of electrical itch stimulation during the SAT in the main analyses for the dot-probe task, we did not find a significant picture position × itch stimulation position interaction (*p* = 0.09), but there was a significant dot position × itch stimulation side interaction (*p* < 0.01), with profile plots indicating that participants that had received itch stimuli on the right arm were slower to respond to the dots on the right side and vice versa for the left side (i.e., slower to respond to the left dots). More importantly, there was no significant itch picture position (left/right) × dot position (left/right) × itch stimulation side interaction (*p* = 0.96) and the main results remained the same (*p* < 0.01), indicating that the itch stimulation side during the SAT did not influence the main findings for the dot-probe. Similarly, for the SAT, there was no significant block type × itch stimulation side interaction (*p* = 0.76), but there was a significant congruency × itch stimulation side interaction (*p* < 0.01) with profile plots indicating that participants stimulated on the left side seemed generally faster incongruently than congruently, whereas reaction times for congruent and incongruent trials in the participants stimulated on the right side seemed roughly comparable. More importantly, there was no significant congruency × block type × itch stimulation side interaction (*p* = 0.58), indicating that the itch stimulation side during the SAT did not influence the main findings for the SAT.

### Relationships between attention tasks and individual characteristics

When exploring the associations between the individual characteristics measured by self-report questionnaires for neuroticism (EPS-RSS), attention (BVS, BSQ-f, PVAQ-A), and catastrophizing (PCS-A) and the attentional bias indices from the three behavioral attention tasks, there were no significant correlation coefficients, except for a significant correlation between more self-reported attention for bodily sensations (BVS) and less attentional bias to itch words in the Stroop task (*r*_s_ = −0.35, *p* = 0.03). The correlation coefficients across the behavioral tasks were all non-significant, i.e., between the modified Stroop task and the dot-probe task (*r*_s_ = −0.02, *p* = 0.91), between the modified Stroop task and SAT (*r*_s_ = −0.11, *p* = 0.52), and between the dot-probe task and SAT (*r*_s_ = −0.02, *p* = 0.93).

## Discussion

Itch-related attentional processing was investigated for the first time, using different behavioral paradigms, which included semantic (modified Stroop), pictorial (dot-probe), and somatosensory (SAT) itch stimuli. Whereas the modified Stroop task and the dot-probe task for itch have the advantage that these are easy to use, and still contain relatively valid representations of itch given its contagious properties, the SAT, using tonic itch stimuli, has the advantage that the use of somatosensory stimuli better approximates the symptoms patients experience clinically. The results indicate that the participants, who were mainly young females, displayed a biased attention toward itch-related information, in both the modified Stroop task and the dot-probe task. The results of the SAT do not point toward biased attention toward the somatosensory itch stimuli. Overall, these findings indicate that attentional processes are also relevant for itch.

The finding that the participants have enhanced attention for itch-related words, in the modified Stroop task, is in line with previous findings of an exploratory study investigating attentional bias for itch words (Van Laarhoven et al., 2016) and studies investigating attentional bias for disease-related words (including itch) in patients with skin disease (Fortune et al., 2003; Willebrand et al., 2002). Results are also consistent with previous pain research in healthy subjects indicating that pain-related words significantly attract more attention than neutral words, particularly when administered in a blocked design (Crombez et al., 2013). Future research should attempt to also include itch words that can be distinguished based on sensory and affective content, since patients with chronic pain have been shown to display attentional bias for sensory pain words, but not for affective pain words (Crombez et al., 2013).

This study also demonstrated for the first time that healthy subjects have an enhanced attention for pictorial itch stimuli in the dot-probe task using 500 ms presentation time of the pictorial stimuli. This is in line with indirect evidence of studies on contagious itch: these studies suggest that people direct attention toward itch and itch-related signals as people feel itch and start scratching while observing others scratching (Schut et al., 2015; Holle et al., 2012). The degree of itch contagion is assumed to be modulated by attention-related gating processes (Holle et al., 2012). Related studies that investigated attentional processing of pain do not generally indicate that healthy subjects have more attention to pain stimuli in the dot-probe task, though the number of studies using a pictorial dot-probe task is restricted (see meta-analysis Crombez et al., 2013). The finding of attentional bias for itch pictures in the present study in contrast to the more inconsistent findings in pain may be explained by the dissimilar reflex pattern of itch and pain. Although both responses have the function of protecting against potential harm (Ikoma et al., 2006), they are characterized by unique responses in their acute state, i.e., scratching in itch and retraction in pain (Ikoma et al., 2006). The reason why we scratch when experiencing itch is unknown and probably results from evolution, as also animals display this unique behavioral response (Handwerker, 2014). Moreover, also the contagiousness of itch may play a role when displaying itch pictures, as it has been shown that itch pictures can induce an itch sensation (Schut et al., 2015), whereas looking at pain pictures often does not lead to pain perception in the observer (Vandenbroucke et al., 2013). Moreover, in contrast to pain that may have a visceral origin, itch is restricted to the skin and adjacent mucosa. For this reason, one might particularly be attentive to (external) stimuli related to itch. These unique and pain-corresponding facets of itch in relation to attentional processes could be best addressed in studies that directly compare attentional processing of itch and pain.

The finding that participants did not display significantly more attention toward somatosensory itch stimuli, in the SAT, using tonic 35-s itch stimuli, is contrary to what was expected. A shift in attention allocation over time, i.e., attentional disengagement during the second time segment of the SAT blocks, may partly explain the lack of significant findings in the overall analyses. Specifically, when taking into account the time course within the blocks, we found that in the second time segment of the itch blocks, participants responded significantly slower to congruent trials than incongruent trials. This suggests that over time participants disengaged attention from the itch location. Compared with studies on pain using somatosensory pain stimuli, it has, for example, been shown that anticipation of pain and experienced pain resulting from phasic stimuli directed attention toward the pain location (i.e., attentional engagement) (Van Damme et al., 2004c, 2007). Also in a study in which participants performed a visual sustained attention task while perceiving a tonic pain stimulus of 10 s, participants performed the sustained attention task faster at the pain location than at the other location, indicating attentional engagement toward the pain location (Van Ryckeghem et al., 2011).

That the participants in the present study may have been able to disengage attention may be explained by the different somatosensory quality of itch versus pain. Pain may generally be appraised as more threatening than itch, associated with increased fear, which in turn increases attention (Crombez et al., 2005; Legrain et al., 2009; Vlaeyen and Linton, 2000; Lazarus, 1993). Also, the design of our study and previous studies differed on some points. For example, as opposed to a sequential order of pain and visual target stimuli in previous studies, in the present study visual targets were given during the itch stimulus, which may have had a distraction effect. The fact that the mean itch levels were significantly higher during the practice stimulus than during the somatosensory attention task (data not shown) supports this notion. Also, the duration of the tonic itch stimulus in the present study (35 s) was at least more than three times longer than in previous studies focusing on pain and attention (Van Ryckeghem et al., 2011), mostly applying phasic rather than tonic somatosensory pain stimuli for their specific research questions (e.g., Spence et al., 2002; Dowman, 2011; Van Damme et al., 2004b, 2007). When aiming to investigate the natural course of attention allocation during somatosensory stimuli, the application of tonic stimuli is required. For the present study, randomization of inter-target intervals prevented temporal alignment of the data further than splitting the blocks into two parts. In future studies, the time course of attention allocation should be assessed more thoroughly as this better approximates the nature of attentional processing (Zvielli et al., 2014). This could reveal information about attentional engagement processes and one’s capacity to disengage attention from symptoms, which is assumed to be disturbed in chronic pain (Crombez et al., 2013; Schoth et al., 2012) and may also be disturbed in chronic itch.

The three behavioral attention tasks seem adequate to measure attentional processing of itch and probably reveal different aspects. Participants displayed an attentional bias for the visual itch representations in the modified Stroop task and dot-probe task, but not for the somatosensory itch stimuli in the SAT. This might particularly be related to the varying modality (verbal, pictorial, somatosensory) and duration of displaying the itch stimuli in the different tasks. Moreover, the absence of significant intercorrelations between the outcomes of the behavioral attention tasks (also not between the two tasks using visual itch stimuli) is in line with previous research in pain (Asmundson, Wright, & Hadjistavropoulos, 2005; Dittmar, Krehl, & Lautenbacher, 2011). It supports the conclusion that the tasks reflect different aspects of attentional itch processing, such as involvement of higher order inhibitory processes or potential involvement of freezing responses for emotional valenced stimuli in the modified Stroop task (Karmann, Lautenbacher, & Kunz, 2015; Nigg, 2000; De Ruiter and Brosschot, 1994).

However, some remaining points related to this study are worth mentioning. First, the sample consisted mainly of female participants. Although we did not find indications that sex influenced the main results, the lack of an equal sex distribution limits the generalizability of findings to the general population (for instance, pain sensitivity has been shown to differ for males and females, e.g., Keogh, 2009; Bartley & Fillingim, 2013). Furthermore, the inclusion criterion of a restricted age range did result in the recruitment of a homogenous group, but it also implies that results cannot directly be extrapolated to another age group. This limitation should be addressed in future research, for instance by including equal proportions of males and females and participants of different age groups to increase the generalizability of the results. Moreover, the questionnaire scores were all relatively low when compared with normative data (Arrindell, 1993; Spinhoven et al., 1997; Roelofs, Peters, Muris, & Vlaeyen, 2002; Sanderman et al., 1995; Van Laarhoven et al., 2010), confirming that the group is healthy and homogenous as intended to exclude other influences. Future research may, however, opt to include a sample from the general population and see whether current findings extend to the whole general population. Second, the predominantly non-significant exploratory correlations (1 out of 15 correlations was significant) between the individual characteristics and attentional bias indices for itch resulting from the behavioral attention tasks has been reported previously (Dittmar et al., 2011). Moreover, the individual difference variables, such as catastrophizing and neuroticism, seem not to play a key role in attentional bias for itch in healthy subjects. Although previous research in healthy subjects incidentally found significant indications for an association between catastrophizing and attentional bias for pain (e.g., Van Damme et al., 2004a, 2007), a meta-analysis indicated that individual difference variables such as fear of pain were not significantly associated with attentional bias for pain (Crombez et al., 2013). Future research has to focus more closely on the relationship between self-reported and behavioral indicators of attentional processing of itch. Third, although the questionnaires assessing catastrophizing (PCS) and vigilance and awareness (PVAQ) are well validated for pain, these had been adjusted for the use in itch; hence, validity remains to be demonstrated. Fourth, during the SAT there were more technical problems than anticipated. Although it is unlikely that results have been biased, because the problems occurred randomly, these may have impacted the statistical power of the SAT analyses. Fifth, the dot-probe task, in contrast to the modified Stroop task, did not have non-itch-related emotional conditions. Including also other affective stimuli in future research with the dot-probe task can enhance the conclusiveness of findings with respect to the attention being related to valence or specific for itch. Lastly, it remains to be investigated whether these tasks are applicable to demonstrate attentional bias for itch-related stimuli in patients with chronic itch. Investigating attentional processing of itch using multiple modalities contributes to our knowledge of the processing of this prevalent, yet understudied symptom.

To conclude, this study for the first time shows that attentional processes also play a role in itch. Moreover, the present study also indicates that attentional itch processing can be measured using behavioral tasks for itch. Both traditionally used tasks (e.g., dot-probe task) and the newly developed somatosensory attention task seem promising measures. These tasks, probably reflecting different aspects of attentional processing also due to differential methodology, may be used to investigate whether patients with chronic itch display attentional bias for itch, similarly to patients with chronic pain who may display attentional bias toward pain (Crombez et al., 2013; Schoth et al., 2012). Moreover, in line with pain (Van Ryckeghem et al., 2013), attentional bias to itch might be used as a predictor for condition-related disability. The use of behavioral attention tasks modified for training purposes is also being explored to train pain-related attention and reduce pain (e.g., Sharpe et al., 2012). For itch, the behavioral tasks using tonic somatosensory or pictorial itch stimuli may have great potential to train possible itch-related attentional bias in patients with chronic itch.
